# Whole-body arginine dimethylation is associated with all-cause mortality in adult renal transplant recipients

**DOI:** 10.1007/s00726-021-02965-1

**Published:** 2021-03-02

**Authors:** Adrian Post, Alexander Bollenbach, Stephan J. L. Bakker, Dimitrios Tsikas

**Affiliations:** 1 Division of Nephrology, Department of Internal Medicine, University Medical Center Groningen and University of Groningen, 9700 RB Groningen, The Netherlands; 2 Core Unit Proteomics, Institute of Toxicology, Hannover Medical School, Carl-Neuberg-Strasse 1, 30625 Hannover, Germany

**Keywords:** Cardiovascular risk, Dimethylation, Kidney, Mortality, Post-translational modification, Transplantation

## Abstract

Arginine residues in proteins can be singly or doubly methylated post-translationally. Proteolysis of arginine-methylated proteins provides monomethyl arginine, asymmetric dimethylarginine (ADMA) and symmetric dimethylarginine (SDMA). ADMA and SDMA are considered cardiovascular risk factors, with the underlying mechanisms being not yet fully understood. SDMA lacks appreciable metabolism and is almost completely eliminated by the kidney, whereas ADMA is extensively metabolized to dimethylamine (DMA), with a minor ADMA fraction of about 10% being excreted unchanged in the urine. Urinary DMA and ADMA are useful measures of whole-body asymmetric arginine-dimethylation, while urinary SDMA serves as a whole-body measure of symmetric arginine-dimethylation. In renal transplant recipients (RTR), we previously found that higher plasma ADMA concentrations and lower urinary ADMA and SDMA concentrations were associated with a higher risk of all-cause mortality. Yet, in this RTR collective, no data were available for urinary DMA. For the present study, we additionally measured the excretion rate of DMA in 24-h collected urine samples of the RTR and of healthy kidney donors in the cohort, with the aim to quantitate whole-body asymmetric (ADMA, DMA) and symmetric (SDMA) arginine-dimethylation. We found that lower DMA excretion rates were associated with higher all-cause mortality, yet not with cardiovascular mortality. In the healthy donors, kidney donation was associated with considerable decreases in ADMA (by − 39%, *P* < 0.0001) and SDMA (by − 21%, *P* < 0.0001) excretion rates, yet there was no significant change in DMA (by − 9%, *P* = 0.226) excretion rate. Our results suggest that protein-arginine dimethylation is altered in RTR compared to healthy kidney donors and that it is pronouncedly shifted from symmetric to asymmetric arginine-dimethylation, with whole-body protein-arginine dimethylation being almost unaffected.

**Supplementary Information:**

The online version contains supplementary material available at 10.1007/s00726-021-02965-1.

## Introduction

**Scheme 1:**
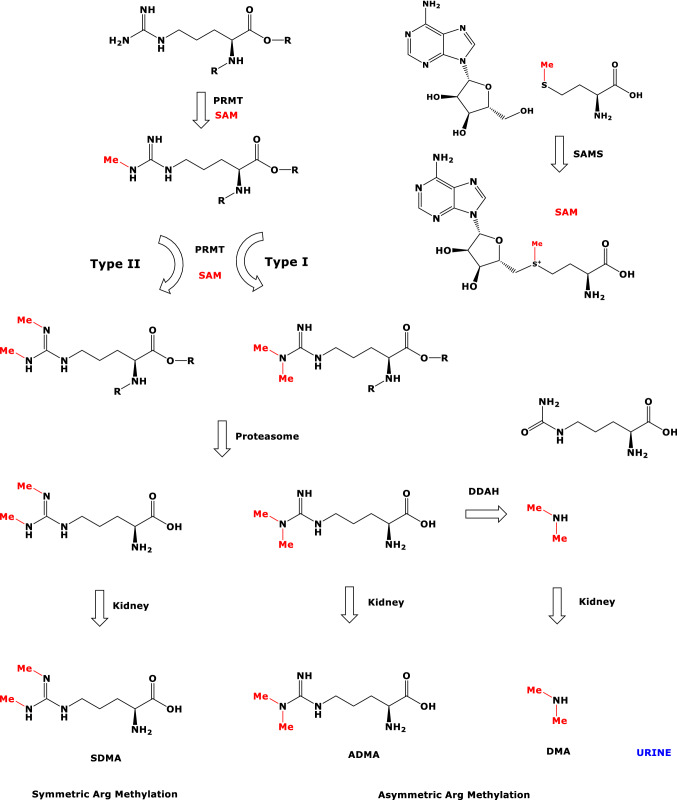
Overview of metabolic routes involved in protein-arginine dimethylation. *PRMT* protein-arginine methyltransferase, *SAM* *S*-adenosyl methionine, *SAMS* *S*-adenosyl methionine synthase, *Me* methyl, *DDAH* dimethylarginine dimethylaminohydrolase, *SDMA*, symmetric dimethylarginine, *ADMA* asymmetric dimethylarginine, *DMA* dimethylamine

l-Arginine (Arg) residues of proteins undergo two major post-translational modifications (PTM), i.e., citrullination and methylation (Scheme 1). Citrullination is catalyzed by Ca^2+^-dependent protein-arginine deiminases (PAD; EC 3.5.3.15). Methylation of the guanidine (*N*^G^) group is catalyzed by protein-arginine methyltransferases (PRMT; EC 2.1.1.319, EC 2.1.1.320), which use the universal methyl-group donor *S*-adenosyl-methionine (SAM) as cofactor. Arg-methylation starts with the formation of *N*^G^-monomethylarginine (MMA) proteins, which are subsequently further methylated to form asymmetric (a) *N*^G^,*N*^G^-dimethylarginine proteins or symmetric (s) *N*^G^,*N*′^G^-dimethylarginine proteins (Blanc and Richard 2017; Peng and Wong 2017) (Scheme 1). The biological significance of authentic asymmetrically protein-arginine dimethylated (aPADiMe) proteins and of symmetrically protein-arginine dimethylated (sPADiMe) proteins in histones and beyond is little investigated and understood. In general, protein-arginine dimethylation (PADiMe) is considered to alter the inherent biological activity of the native proteins (Blanc and Richard 2017; Peng and Wong 2017; Greer and Shi 2012; Beltran-Alvarez et al. 2011, 2014, 2015; Samuel et al. 2021; Sirover 2021). PTM may also be considered as a source of metabolites that are involved in various pathways.

Proteolysis of Arg-methylated proteins generates free *N*^G^-monomethylarginine (MMA), asymmetric *N*^G^,*N*^G^-dimethylarginine (ADMA) and symmetric *N*^G^,*N*′^G^-dimethylarginine (SDMA). MMA, ADMA and SDMA are endogenous inhibitors of the activity of nitric oxide synthase (NOS; EC 1.14.13.39) isoforms (Tsikas 2008, 2017; Tsikas et al. 2018). NOS converts free l-arginine (Arg) to l-citrulline and nitric oxide (NO). NO is a pleiotropic signaling molecule and one of the most potent endogenous vasodilators and regulators of blood pressure (Tousoulis et al. 2012). Elevated circulating ADMA concentrations are associated with hypertension (Achan et al. 2003). ADMA and SDMA are considered cardiovascular risk factors and have emerged as predictors of cardiovascular events and death in a range of pathologies, including kidney disease (Busch et al. 2006; Tain and Hsu 2017) and kidney transplantation (Frenay et al. 2015a, b; Said et al. 2019a, b).

MMA and ADMA are hydrolyzed mainly by hepatic and renal dimethylarginine dimethylaminohydrolase (DDAH; EC 3.5.3.18) to form monomethyl amine and dimethyl amine (DMA), respectively, while SDMA is not metabolized by DDAH (Scheme 1). MMA, ADMA, SDMA and DMA circulate in blood at nM-to-µM concentrations and are excreted in the urine (Tsikas 2008; Martens-Lobenhoffer and Bode-Böger 2014). In adults, up to 90% of daily produced ADMA is estimated to be hydrolyzed to DMA, with the remaining 10% of ADMA being excreted unchanged in the urine (Tsikas 2020). Thus, urinary DMA is the major urinary metabolite of ADMA in humans.

Measurement of major urinary metabolites of circulating precursors is a widely used non-invasive approach to estimate their whole-body synthesis of endogenous substances such as DMA (Achan et al. 2003; Tsikas 2020 and references therein), nitrate (Baylis and Vallance 1998), prostaglandins (Ferretti et al. 1983), thromboxane (Roberts et al. 1977), leukotrienes (reviewed in Tsikas 1998), RNA (Sander et al. 1986), and catecholamines (Duncan et al. 1988). Recently, we proposed such a method to measure whole-body PADiMe activity in humans and demonstrated its utility in human studies (Bollenbach et al. 2019; Bollenbach et al. 2020a, b). PADiMe activity indices (PADiMeX) include urinary DMA, ADMA, SDMA and their composites, with the index DMA + ADMA reflecting asymmetric PADiMe activity (aPADiMeX), the index DMA + ADMA + SDMA reflecting total PADiMe activity (toPADiMeX), and the index (DMA + ADMA)/SDMA reflecting the balance between asymmetric and symmetric PADiMe activities (a/sPADiMeX) (Bollenbach et al. 2019).

In the present study, we measured the excretion of DMA in urine samples of renal transplant recipients (RTR) and healthy kidney donors, in which we had already measured the excretion of ADMA and SDMA (Said et al. 2019a, b). Using these data, we applied the PADiMe approach and investigated its potential associations with long-term mortality in RTR. Our study is the first to report on the whole-body protein-arginine dimethylation in RTR and in healthy donors prior to and after kidney donation.

## Experimental

### Design and study population

Study design has been described in detail (Post et al. 2018; van den Berg et al. 2012, 2013, 2014) and is part of a larger prospective cohort study of RTR in northern Netherlands (Transplantlines Food and Nutrition cohort, Clinicaltrials. gov no. NCT02811835). In the present study both RTR and living kidney donors participated. All of them gave their informed consent and all transplantations were conducted at the University Medical Center Groningen (UMCG). The main inclusion criterion was having a renal graft that had been functioning for at least 1 year. Main exclusion criteria were drug and alcohol abuse, overt congestive heart failure (NYHA 3–4), malignancy (other than cured skin cancer) and an insufficient understanding of the Dutch language. The baseline examination of each participant was performed between November 2008 and March 2011 and participants were followed up until the end of August 2015. The data of 691 RTR were included for statistical analyses. A total of 121 kidney donors had pre-donation data available and a total of 97 kidney donors had post-donation data available. Out of these kidney donors, there were 76 kidney donors with data available both pre- and post-donation. The study protocol (METc 2008/186) was approved by the institutional ethical review board of the UMCG and has been conducted in accordance with the declaration of Helsinki. The primary outcome measure of the study was all-cause mortality. Secondary endpoints were cardiovascular mortality and death-censored graft loss (defined as return to dialysis or re-transplantation).

### Clinical measurements

According to instructions, each participant collected 24-h urine on the day prior to their visit to the outpatient clinic. After an overnight fasting period, all participants (included RTR and healthy kidney donors) visited the outpatient clinic in the morning. Anthropometric measurements were performed on the same day as blood and urine collection. A strict protocol was followed for the measurements of blood pressure (mmHg) and heart rate with a semi-automatic device (Dinamap^®^ 1846, Critikon, Tampa, FL, USA) every minute for the duration of 15 min, the final value was defined as the average of the last three values. Detailed descriptions of anthropometric measurements have been described before (van den Berg et al. 2012, 2013, 2014). For routine clinical chemistry assays, heparin plasma was analyzed spectrophotometrically on the same morning using automated and validated routine methods (Roche Diagnostics, Basel, Switzerland).

### Measurement of urinary DMA, ADMA and SDMA

The urine samples were transferred from UMCG frozen on dry ice to the Institute of Toxicology at Hannover Medical School and stored there (at − 20 °C) until analysis. DMA was newly measured in 10-µL urine aliquots by GC–MS in the positive ion chemical ionization mode after extractive derivatization with pentafluorobenzoyl chloride (Merck, Darmstadt, Germany) as described elsewhere with minor modifications (Tsikas et al. 2007). Hexadeutero-dimethylamine (d_6_-DMA; Sigma-Aldrich, Germany) was used as internal standard and added to the urine samples at a final concentration of 1 mM. Study urine samples were analyzed for DMA within nine runs alongside urine quality control (QC) samples which were analyzed in duplicate. Ions with mass-to-charge (*m*/*z*) ratios of *m*/*z* 240 for endogenous DMA (d_0_-DMA) and *m*/*z* 246 for the internal standard (d_6_-DMA) were used in the selected-ion monitoring (SIM) mode. The analytical performance of the GC–MS method for urinary DMA in the present study is reported in “Results”. ADMA (Tsikas et al. 2003) and SDMA (Bollenbach et al. 2018) were measured by GC–MS/MS and GC–MS in 10-µL urine aliquots after derivatization with methanolic 2 M HCl and pentafluoropropionic anhydride as described previously, respectively.

### Statistical analyses

Data analyses and computations were performed with SPSS 24.0 software (IBM, Armonk, NY, USA), Stata SE version 15 (StataCorp, College Station, TX, USA), R version 3.5.1 software (The R-Foundation for Statistical Computing), and GraphPad Prism version 5 (GraphPad Software).

Baseline data are presented as means ± standard deviation for normally distributed data, as medians (interquartile range) for non-normally distributed data, and as numbers (percentages) for nominal data. A two-sided *P* value < 0.05 was considered to indicate statistical significance. In our first analyses, we aimed to investigate the effect of kidney donation on changes in the urinary excretion rates of DMA, ADMA and SDMA. Therefore, in these analyses, we included only kidney donors with both pre- and post-donation data available (*n* = 76). Second, we aimed to compare the data of kidney donors to data of RTR. Therefore, we included a total of 121 kidney donors with pre-donation data available and a total of 97 kidney donors with post-donation data available. Differences in baseline variables amongst sex-stratified tertiles of urinary DMA excretion were studied using ANOVA, Kruskal–Wallis tests or Chi-squared tests. In supplementary analyses, we employed linear regression analyses to investigate the association of plasma ADMA with urinary ADMA excretion and the association of plasma ADMA with urinary DMA excretion.

Prospective analyses of urinary DMA excretion, urinary DMA and ADMA excretion, urinary DMA, ADMA and SDMA excretion and the ratio between urinary DMA and ADMA to urinary SDMA were performed for all-cause mortality, cardiovascular mortality and non-cardiovascular mortality. The continuous surveillance system of the outpatient program ensured that there was up-to-date information on patient status. Endpoints were recorded until September 2015 by a qualified physician. There was no loss that was due to follow-up for the primary endpoints. Prospective analyses were performed using uni- and multivariable Cox regression models. Listwise deletion was used to assure that the number of cases and participants was equal among the models. Adjustments were made for a priori selected variables and for potentially relevant variables identified from the baseline table by a *P* value < 0.05. A priori-selected variables were basic potential confounders, including age, sex, body mass index (BMI), estimated glomerular filtration rate (eGFR) and proteinuria (model 3). To avoid overfitting and inclusion of too many variables for the number of events, additional models were created using additive adjustments to model 3. In the subsequent models, we adjusted for cardiovascular risk factors, transplantation-related factors and primary renal disease. Cardiovascular risk factors were defined as total cholesterol, HDL cholesterol, systolic blood pressure, diastolic blood pressure, antihypertensive treatment, smoking, fasting plasma glucose and the presence of diabetes. Transplantation related factors were defined as donor type (deceased versus living), dialysis vintage, time between transplantation and baseline, cold ischemia time, calcineurin inhibitor usage, proliferation inhibitor usage and the number of transplantations up to baseline. The proportionality of hazards assumption was tested with the Schoenfeld residual test and was not violated for the associations of urinary DMA excretion, urinary DMA and ADMA excretion, urinary DMA, ADMA and SDMA excretion and the ratio between urinary DMA and ADMA to urinary SDMA with all-cause mortality, cardiovascular mortality and non-cardiovascular mortality (*P* > 0.05 for all). Potential interactions for covariates were assessed by calculating interaction term, *P* interaction < 0.05 was considered to indicate significant effect-modification. To visualize the continuous associations of urinary DMA excretion, urinary DMA and ADMA excretion, urinary DMA, ADMA and SDMA excretion and the ratio between urinary DMA and ADMA to urinary SDMA with all-cause mortality, cardiovascular mortality and non-cardiovascular mortality, log_2_-transformed urinary DMA excretion, urinary DMA and ADMA excretion, urinary DMA, ADMA and SDMA excretion and the ratio between urinary DMA and ADMA to urinary SDMA, as continuous variables, were plotted against the risk of all-cause mortality, cardiovascular mortality and non-cardiovascular mortality.

## Results

### Performance of the GC–MS method for urinary DMA

**Fig. 1:**
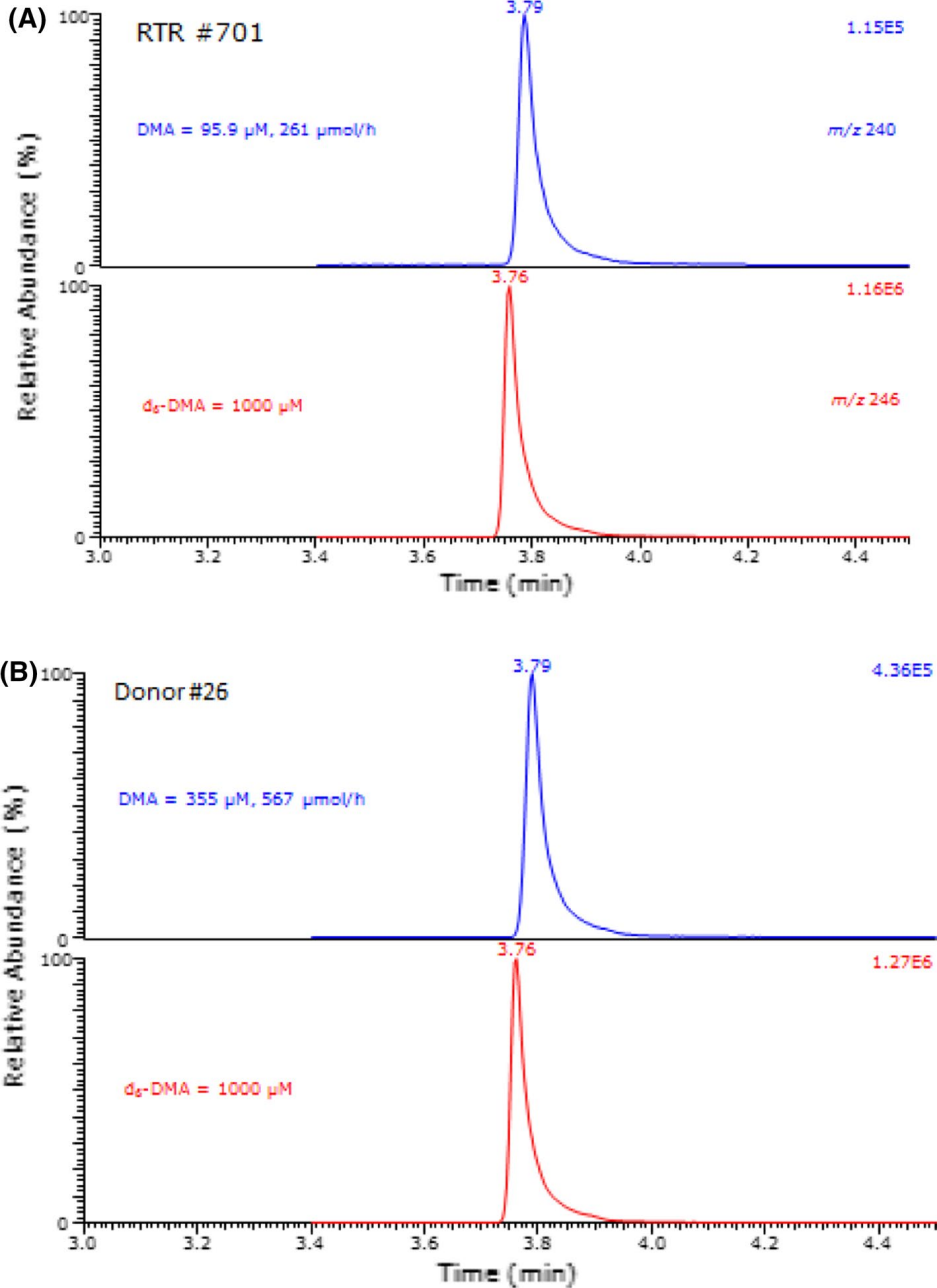
Partial GC–MS chromatograms from quantitative analyses of dimethylamine (DMA) in urine samples of a renal transplant recipient (**a**) and of a healthy subject before donation (**b**). The upper and lower chromatograms show the ion currents produced by ions with *m*/*z* 240 (endogenous DMA) and *m*/*z* 246 (internal standard, d_6_-DMA; 1000 µM), respectively. The retention time of the pentafluorobenzamide derivatives of DMA and d_6_-DMA is 3.79 min and 3.76 min, respectively

The GC–MS method used for the measurement of DMA in the urine samples of the present study had been published previously by our group (Tsikas et al. 2007). Given the particular importance of DMA for the present study, the GC–MS method for urinary DMA was re-evaluated by one of the authors of this article who analyzed all study samples. The method was validated using a urine sample donated by a healthy volunteer at pathophysiologically relevant DMA concentrations of 0, 200, 400, 600, 800 and 1000 µM (reviewed in Tsikas 2020). The nominal concentration of the internal standard d_6_-DMA added to the urine samples was 1000 µM. Analyses in method validation were performed in triplicate for unspiked and spiked DMA concentrations. The precision of the method in terms of relative standard deviation (RSD) ranged between 1.73% and 3.25%. Linear regression analysis between the peak area ratio (PAR) of *m*/*z* 240 to *m*/*z* 246 (*y*) and the added DMA concentration (*x*) resulted in the regression equation *y* = 140 + 1.28*x* (*r*^2^ = 0.9993). The *y*-axis intercept indicates a urinary basal DMA concentration of 140 µM. The accuracy of the method was calculated to range between 109 and 120% for the added DMA concentration. In the QC urine samples, DMA was measured at (mean ± SD, *n* = 9) 138 ± 1.93 µM, i.e., with an inter-assay precision (RSD) of 1.4%. These data underline the high analytical reliability of the GC–MS method for urinary DMA used in the present study. Given the relatively long time period required to perform the large number of DMA analyses for the study and the need to analyze already derivatized urine samples at later time points, three randomly selected urine samples of the study were derivatized and analyzed immediately (day zero) as well as 6 and 16 days thereafter and separately tested for stability over time. The coefficient of variation of these stability analyses was 1.3% at 151 µM, 1.3% at 227 µM and 1.1% at 269 µM. These results indicate long-term stability of the pentafluorobenzamide derivatives of endogenous DMA and the internal standard in the toluene extracts. Typical partial GC–MS chromatograms from quantitative measurements of DMA in urine samples of the present study are shown in Fig. 1.

### Urinary excretion rates of DMA, ADMA and SDMA in healthy kidney donors before and after kidney donation

The anthropometric and clinical characteristics of the donors prior to donation and on average 1.6 months after donation are summarized in Table S1 (see Supplement).

A total of 76 kidney donors had both pre- and post-donation data available. The results of the measurements of the PADiMeX and their statistical comparison are summarized in Table 1. Donation of a kidney did not result in a significant decrease of the DMA excretion rate (430 vs. 390 µmol/24 h, *P* = 0.226), suggesting no appreciable change in the whole-body asymmetric protein Arg-dimethylation due to kidney donation. However, the sum of DMA, ADMA and SDMA decreased significantly by 12% (*P* = 0.006). The decreases in the excretion rates of ADMA (− 38.6%) and SDMA (− 20.7%) resulted in an increase of the (DMA + ADMA)/SDMA ratio from 7.72 to 8.74, suggesting a relative shift from symmetric dimethylation toward asymmetric Arg-dimethylation by about 12%. The urinary excretion rates (in µmol/24 h) of DMA, ADMA and SDMA correlated (*P* < 0.0001) positively among each other before and after kidney donation (Table S2).

**Table 1:** Urinary excretion rates of DMA, ADMA, SDMA and their composites, their ratios, and changes in the healthy kidney donors prior to and after donation

Urinary excretion rate (µmol/24 h) or ratio	Pre-donation (*n* = 76)	Post-donation (*n* = 76)	Post–pre change (%)	*P* value^a^
DMA	430 [357–492]	390 [308–492]	− 9.3	0.2255
ADMA	58.3 [48.6–69.5]	35.8 [31.2–44.8]	− 38.6	** < 0.0001**
SDMA	62.4 [48.5–71.9]	49.5 [37.7–63.8]	− 20.7	** < 0.0001**
DMA + ADMA	479 [415–523]	429 [342–532]	− 10.4	0.0781
DMA + ADMA + SDMA	543 [455–641]	478 [392–604]	− 12.0	**0.0064**
(DMA + ADMA)/SDMA	7.72 [6.81–9.21]	8.74 [7.95–10.82]	+ 11.7	**0.0013**
SDMA/ADMA	1.05 [0.93–1.18]	1.35 [1.03–1.64]	+ 22.2	** < 0.0001**

Data are presented as median [IQR]. A total of 76 kidney donors had both pre- and post-donation data available

Bold numbers indicate statistical significance (*P* value < 0.05)

^a^Statistical analysis was performed using paired *t* test and Wilcoxon rank test when appropriate

### Urinary excretion rates of DMA, ADMA and SDMA in healthy kidney donors and renal transplant recipients

We measured the urinary excretion rates of DMA, ADMA and SDMA in 121 donors prior to donation, in 97 donors after donation, and in 691 or 705 RTR. The data of these analyses and the results of inter-comparisons are summarized in Table 2. The median DMA excretion rate in 691 RTR (429 µmol/24 h) was closely comparable with the DMA excretion rate in the 121 kidney donors before (428 µmol/24 h) and in 97 kidney donors after donation (411 µmol/24 h). The kidney donation resulted in decreases in ADMA and SDMA excretion rates and in increased similarity between RTR and donors after kidney donation. The results presented in Table 2 suggest that RTR have an altered protein-arginine dimethylation state and that donation of a kidney by the healthy donors alters whole-body protein-arginine dimethylation to a degree comparable to that in the RTR patients.

**Table 2:** Urinary excretion rates of DMA, ADMA, SDMA and their composites, their ratios in the healthy kidney donors before and after donation and in the renal transplant recipients (RTR) and their percentage differences

Urinary excretion rate (µmol/24 h) or ratio	Pre-donation (*n* = 121)	Post-donation (*n* = 97)	RTR (*n* = 691)	Difference RTR-pre (%)	Difference RTR-post (%)	*P* value^a^	
	A	B	C	C–A	C–B	A vs. C	B vs. C
DMA	426 [360–480]	411 [327–515]	429 [353–516]	0.7	4.2	0.41	0.19
ADMA	60 [49–70]	36 [31–46]	30 [22–40]	− 49.3	− 15.1	** < 0.001**	** < 0.001**
SDMA	61 [50–70]	50 [38–64]	50 [40–63]	− 18.3	0.8	** < 0.001**	0.88
DMA + ADMA	490 [419–536]	440 [361–560]	460 [381–551]	− 6.1	+ 4.3	**0.05**	0.40
DMA + ADMA + SDMA	550 [482–613]	498 [402–628]	513 [425–609]	− 6.7	+ 2.9	**0.02**	0.47
(DMA + ADMA)/SDMA	8.2 [7.1–9.3]	8.8 [8.0–10.8]	9.0 [7.8–10.9]	+ 9.4	+ 2.3	** < 0.001**	0.69
SDMA/ADMA	1.03 [0.90–1.17]	1.33 [1.08–1.63]	1.71 [1.35–2.17]	+ 39.8	+ 22.2	** < 0.001**	** < 0.001**

Data are presented as median [IQR]. To obtain maximal statistical power for our analyses, we did not only include kidney donors with both pre- and post-donation data available, but also included participants with either pre-donation or post-donation data available. Therefore, the number of kidney donors is 121 pre-donation and 97 post-donation

Bold numbers indicate statistical significance (*P* value < 0.05)

^a^Statistical analyses were performed between A and C, and between B and C using the Kruskal–Wallis test

In the RTR, the urinary excretion rate of DMA (µmol/24 h) correlated positively with the urinary excretion rates (µmol/24 h) of ADMA (*r* = 0.399) and SDMA (*r* = 0.550), with ADMA and SDMA also correlating with each other (*r* = 0.573). All these Spearman correlations were significant (*P* < 0.0001) (Table S3).

### Cohort characteristics and urinary Arg-dimethylation biomarkers

In the whole cohort of 691 RTR (age, 53 ± 13 years; gender, 57% male; eGFR, 45 ± 19 mL/min/1.73 m^2^), the median [IQR] urinary DMA excretion was 429 [353–516] μmol/24 h. Median [IQR] urinary ADMA and SDMA excretion in RTR were 30 [22–40] μmol/24 h and 50 [40–63] μmol/24 h, respectively. The ratio of the sum of DMA and ADMA to SDMA, i.e., (DMA + ADMA)/SDMA, the asymmetric-to-symmetric protein-arginine dimethylation index a/sPADiMeX, was calculated to be 9.0 [7.8–10.9]. Differences in protein-arginine dimethylation and differences in baseline characteristics amongst sex-stratified tertiles of urinary DMA excretion are summarized in Table 3. Patients in the lowest sex-stratified tertile of urinary DMA excretion had higher HDL-cholesterol and more time between baseline and transplantation (all *P* < 0.05). In contrast, patients in the lowest sex-stratified tertile of urinary DMA excretion had lower weight, height, BMI, body surface area (BSA), diastolic blood pressure, urinary sodium excretion, and less frequent usage of inhibitors of calcineurin, a calcium- and calmodulin-dependent serine/threonine protein phosphatase (all *P* < 0.05). In Table S4, we investigate the associations of plasma ADMA concentration with urinary ADMA excretion and urinary DMA excretion. After adjusting for age, sex, BMI and eGFR, there was a strong positive association between plasma ADMA concentration and urinary ADMA excretion (Std. *β*: 0.36; *P* < 0.001). No association was found between plasma ADMA concentration and urinary DMA excretion.

**Table 3:** Baseline characteristics according to sex-stratified tertiles of DMA

	RTR cohort (*n* = 691)	Tertile 1 (*n* = 230)	Tertile 2 (*n* = 231)	Tertile 3 (*n* = 230)	*P* value
Arg-dimethylation indices					
DMA (μmol/24 h)	429 [353–516]	331 [301–395]	447 [370–486]	553 [454–618]	** < 0.001**
ADMA (μmol/24 h)	30 [22–40]	26 [18–32]	32 [25–41]	34 [26–44]	** < 0.001**
SDMA (μmol/24 h)	50 [40–63]	42 [34–50]	52 [44–63]	58 [45–71]	** < 0.001**
DMA + ADMA (μmol/24 h)	460 [381–551]	358 [326–426]	479 [399–521]	587 [488–657]	** < 0.001**
DMA + ADMA + SDMA (μmol/24 h)	513 [425–609]	403 [361–478]	533 [444–581]	651 [554–723]	** < 0.001**
(DMA + ADMA)/SDMA	9.0 [7.8–10.9]	8.5 [7.5–10.4]	8.8 [7.7–10.1]	10.0 [8.4–12.7]	** < 0.001**
Demographics					
Age (years)	53 ± 13	54 ± 13	53 ± 12	52 ± 12	0.30
Sex [*n* (% male)]	391 (57)	130 (57)	131 (57)	130 (57)	0.99
Smokers [*n* (%)]					
Never	270 (42)	87 (40)	95 (44)	88 (41)	**0.02**
Past	298 (46)	107 (49)	104 (48)	87 (41)	
Current	81 (12)	24 (11)	18 (8)	39 (18)	
Alcohol					
0–10 g/24 h	463 (73)	160 (75)	143 (69)	160 (76)	0.26
10–30 g/24 h	137 (22)	46 (22)	50 (24)	41 (19)	
> 30 g/24 h	30 (5)	6 (3)	14 (7)	10 (5)	
Body composition					
Weight (kg)	80 ± 17	73 ± 14	81 ± 14	87 ± 18	** < 0.001**
Height (cm)	174 ± 10	171 ± 10	175 ± 9	175 ± 10	** < 0.001**
BMI (kg/m^2^)	26.6 ± 4.8	25.0 ± 4.1	26.6 ± 4.4	28.2 ± 5.3	** < 0.001**
BSA (m^2^)	1.94 ± 0.22	1.84 ± 0.20	1.96 ± 0.19	2.02 ± 0.23	** < 0.001**
Primary renal disease *n* (%)					
Primary glomerulosclerosis	195 (28)	61 (27)	69 (30)	65 (28)	0.73
Glomerulonephritis	53 (8)	17 (7)	18 (8)	18 (8)	0.98
Tubulointerstitial nephritis	84 (12)	32 (14)	24 (10)	28 (12)	0.51
Polycystic kidney disease	144 (21)	44 (19)	47 (20)	53 (23)	0.57
Hypo- or dysplasia	28 (4)	11 (5)	14 (6)	3 (1)	**0.03**
Renovascular disease	36 (5)	15 (6)	11 (5)	10 (4)	0.54
Diabetes	35 (5)	14 (6)	9 (4)	12 (5)	0.56
Cardiovascular parameters					
Systolic blood pressure (mmHg)	136 ± 18	135 ± 17	138 ± 18	136 ± 18	0.22
Diastolic blood pressure (mmHg)	83 ± 11	81 ± 11	84 ± 11	83 ± 11	**0.03**
Mean arterial pressure (mmHg)	107 ± 15	106 ± 15	109 ± 15	107 ± 16	0.12
Pulse pressure (mmHg)	53 ± 13	106 ± 15	109 ± 15	107 ± 16	0.72
Heart rate (bpm)	69 ± 12	54 ± 13	54 ± 14	53 ± 12	0.39
Hypertension^a^	284 (41)	88 (39)	107 (46)	89 (39)	0.16
Antihypertensive drugs [*n* (%)]	609 (88)	198 (86)	209 (90)	202 (88)	0.34
NT-proBNP (ng/L)	255 [109–638]	263 [99–759]	245 [113–557]	261 [111–619]	0.49
Urinary sodium excretion (mmol/24 h)	157 ± 62	132 ± 53	159 ± 54	181 ± 68	** < 0.001**
Lipids					
Total cholesterol (mM)	5.1 ± 1.1	5.2 ± 1.2	5.0 ± 1.1	5.1 ± 1.1	0.12
HDL cholesterol (mM)	1.4 ± 0.5	1.5 ± 0.5	1.4 ± 0.5	1.3 ± 0.4	**0.007**
LDL cholesterol (mM)	3.0 ± 0.9	3.0 ± 1.0	2.9 ± 0.9	3.0 ± 0.9	0.41
Triglycerides (mM)	1.7 [1.2–2.3]	1.6 [1.2–2.3]	1.7 [1.2–2.2]	1.7 [1.3–2.3]	0.65
Statins [*n* (%)]	365 (53)	126 (55)	127 (55)	112 (49)	0.31
Glucose homeostasis					
Glucose (mM)	5.7 ± 1.7	5.8 ± 1.9	5.4 ± 1.1	5.8 ± 1.9	**0.01**
HbA_1c_ (%)	5.8 [5.5–6.2]	5.8 [5.5–6.2]	5.8 [5.5–6.1]	5.7 [5.5–6.3]	0.92
Diabetes [*n* (%)]	158 (23)	57 (25)	40 (17)	61 (27)	**0.04**
Antidiabetic drugs [*n* (%)]	102 (15)	36 (16)	29 (13)	37 (16)	0.51
Transplantation-related					
Dialysis vintage (m)	27 [9–52]	23 [8–48]	28 [10–53]	30 [10–54]	0.26
Time since kidney transplantation (*y*)	5.5 [1.9–12.1]	7.3 [3–15]	5.7 [2.3–12.1]	4.2 [1.2–10.0]	** < 0.001**
Deceased donor [*n* (%)]	458 (66)	156 (68)	147 (64)	155 (67)	0.58
Cold ischemia time (h)	15 [3–21]	17 [3–23]	14 [3–21]	15 [3–21]	0.18
Warm ischemia time (min)	43 ± 15	43 ± 16	42 ± 14	44 ± 15	0.56
Transplantations up to baseline					
1 transplantation [*n* (%)]	623 (90)	203 (88)	210 (91)	210 (91)	0.49
≥ 2 transplantations [*n* (%)]	68 (10)	27 (12)	21 (9)	20 (9)	
Calcineurin inhibitors [*n* (%)]	395 (57)	119 (52)	129 (56)	147 (64)	**0.03**
Proliferation inhibitors [*n* (%)]	573 (83)	186 (81)	193 (84)	194 (84)	0.58
HLA antibodies [*n* (%)]					
HLA-I	103 (15)	32 (14)	32 (14)	39 (17)	0.57
HLA-II	117 (17)	43 (19)	37 (16)	37 (16)	0.68
Renal function					
Serum creatinine (µM)	125 [100–161]	124 [98–173]	124 [102–154]	127 [100–165]	0.76
eGFR (mL/min/1.73 m^2^)	45 ± 19	45 ± 20	46 ± 17	44 ± 19	0.71
Proteinuria [*n* (%)]	154 (22)	44 (19)	51 (22)	59 (26)	0.24
Venous parameters					
Sodium (mM)	141 ± 3	140 ± 3	141 ± 3	141 ± 3	**0.001**
Albumin (g/L)	43 ± 3	43 ± 3	43 ± 3	43 ± 3	0.57
hs-CRP (mg/L)	1.6 [0.7–4.5]	1.5 [0.7–4.4]	1.6 [0.7–3.4]	1.7 [0.8–5.4]	0.26

Data are presented as mean ± SD, median [IQR] or number (percentage)

Statistical analysis was performed using ANOVA, Kruskal–Wallis or *χ*^2^ test

Bold numbers indicate statistical significance (*P* value < 0.05)

^a^Hypertension is defined as SBP > 140 and/or DBP > 90

### Prospective analyses

During a median follow-up time of 5.4 [4.8–6.06] years, 148 patients died, of which 59 (40%) of cardiovascular causes and 89 (60%) of non-cardiovascular causes. Univariable and multivariable Cox regression analyses of log_2_-transformed urinary DMA excretion, the sum urinary DMA and + ADMA (DMA + ADMA) excretion, the sum of urinary DMA, ADMA and SDMA (DMA + ADMA + SDMA) excretion and the ratio of the sum of urinary DMA and ADMA to urinary SDMA, (DMA + ADMA)/SDMA, with all-cause mortality, cardiovascular mortality and non-cardiovascular mortality are summarized in Table 4. Listwise deletion was employed to keep the number of participants and events similar across the models. Univariable, urinary DMA excretion was inversely associated with all-cause mortality (hazard ratio (HR): 0.65 [0.42–0.99]; *P* = 0.04) and non-cardiovascular mortality (HR: 0.49 [0.29–0.82]; *P* = 0.008). Similarly, the sum of urinary DMA and ADMA excretion (DMA + ADMA) was inversely associated with all-cause mortality (HR: 0.58 [0.38–0.88]; *P* = 0.01) and non-cardiovascular mortality (0.44 [0.26–0.74]; *P* = 0.003). The sum of urinary DMA, ADMA and SDMA excretion (DMA + ADMA + SDMA) was also inversely associated with all-cause mortality (HR: 0.53 [0.34–0.81]; *P* = 0.003) and non-cardiovascular mortality (HR: 0.40 [0.24–0.68]; *P* < 0.001). The associations of urinary DMA excretion, urinary DMA + ADMA excretion and urinary DMA + ADMA + SDMA excretion with all-cause mortality and non-cardiovascular mortality were independent of potential confounders, including age, sex, BMI, eGFR, proteinuria, cardiovascular risk factors, transplantation related factors and primary renal disease (models 2–6). The ratio (DMA + ADMA)/SDMA was positively associated with all-cause mortality (HR: 1.59 [1.17–2.17]; *P* = 0.003) and cardiovascular mortality (HR: 1.76 [1.11–2.79]; *P* = 0.02) in the univariable model. However, the associations were lost after adjustment for potential confounders. Graphical representations of the Cox-regression analyses and Kaplan–Meier curves for urinary DMA and its composites are illustrated in Figs. S1 and S2 (see Supplement).

**Table 4:** Association of urinary excretion rates of DMA, DMA + ADMA, DMA + ADMA + SDMA and (DMA + ADMA)/SDMA with all-cause mortality, cardiovascular mortality, and non-cardiovascular mortality

	All-cause mortality		Cardiovascular mortality		Non-cardiovascular mortality	
	HR per doubling [95% CI]	*P*	HR per doubling [95% CI]	*P*	HR per doubling [95% CI]	*P*
DMA						
Model 1	0.65 [0.42–0.99]	0.04	1.00 [0.52–1.93]	0.99	0.49 [0.29–0.82]	0.008
Model 2	0.58 [0.36–0.93]	0.02	0.72 [0.33–1.57]	0.41	0.51 [0.28–0.90]	0.02
Model 3	0.56 [0.36–0.87]	0.01	0.68 [0.32–1.43]	0.31	0.49 [0.28–0.85]	0.01
Model 4	0.52 [0.33–0.83]	0.006	0.59 [0.27–1.28]	0.18	0.48 [0.28–0.85]	0.01
Model 5	0.56 [0.35–0.88]	0.01	0.66 [0.31–1.40]	0.28	0.48 [0.28–0.95]	0.01
Model 6	0.62 [0.39–1.00]	0.05	0.74 [0.34–1.65]	0.47	0.56 [0.31–1.01]	0.05
DMA + ADMA						
Model 1	0.58 [0.38–0.88]	0.01	0.90 [0.46–1.77]	0.76	0.44 [0.26–0.74]	0.003
Model 2	0.51 [0.32–0.80]	0.004	0.62 [0.29–1.34]	0.23	0.45 [0.26–0.78]	0.005
Model 3	0.54 [0.34–0.84]	0.007	0.65 [0.31–1.38]	0.26	0.48 [0.28–0.83]	0.009
Model 4	0.50 [0.32–0.80]	0.004	0.56 [0.25–1.23]	0.15	0.47 [0.27–0.82]	0.008
Model 5	0.54 [0.34–0.86]	0.008	0.64 [0.30–1.36]	0.25	0.47 [0.27–0.83]	0.009
Model 6	0.60 [0.37–0.97]	0.04	0.71 [0.32–1.59]	0.41	0.54 [0.30–0.98]	0.04
DMA + ADMA + SDMA						
Model 1	0.53 [0.34–0.81]	0.003	0.81 [0.40–1.62]	0.55	0.40 [0.24–0.68]	< 0.001
Model 2	0.46 [0.30–0.72]	< 0.001	0.55 [0.26–1.18]	0.12	0.42 [0.24–0.71]	0.001
Model 3	0.50 [0.32–0.79]	0.003	0.60 [0.28–1.26]	0.17	0.46 [0.26–0.78]	0.005
Model 4	0.47 [0.30–0.74]	0.001	0.51 [0.23–1.11]	0.09	0.45 [0.26–0.78]	0.005
Model 5	0.51 [0.32–0.81]	0.004	0.60 [0.28–1.26]	0.18	0.45 [0.25–0.79]	0.005
Model 6	0.56 [0.35–0.91]	0.02	0.65 [0.29–1.45]	0.29	0.51 [0.28–0.93]	0.03
(DMA + ADMA)/SDMA						
Model 1	1.59 [1.17–2.17]	0.003	1.76 [1.11–2.79]	0.02	1.47 [0.97–2.23]	0.07
Model 2	1.50 [1.09–2.07]	0.01	1.60 [0.99–2.58]	0.05	1.42 [0.93–2.19]	0.11
Model 3	1.33 [0.94–1.90]	0.11	1.48 [0.87–2.50]	0.14	1.23 [0.77–1.96]	0.39
Model 4	1.36 [0.96–1.95]	0.09	1.53 [0.91–2.60]	0.11	1.24 [0.77–2.01]	0.38
Model 5	1.19 [0.83–1.71]	0.35	1.34 [0.78–2.31]	0.29	1.04 [0.64–1.67]	0.88
Model 6	1.31 [0.92–1.87]	0.14	1.46 [0.87–2.48]	0.16	1.19 [0.74–1.93]	0.47
Patients	619		619		619	
Events	128		51		77	

Model 1: Crude model

Model 2: Model 1 + age, sex and BMI

Model 3: Model 2 + eGFR and proteinuria

Model 4: Model 3 + cardiovascular risk factors (total cholesterol, HDL cholesterol, systolic blood pressure, antihypertensive treatment, smoking (current, ex, or never) and diabetes)

Model 5: Model 3 + transplantation related factors (donor type, total dialysis time, time from transplantation to baseline, cold ischemia time, CNI usage, proliferation inhibitor usage and transplantation count)

Model 6: Model 3 + urinary excretion of sodium

eGFR was calculated according to the chronic kidney disease epidemiology formula with plasma creatinine and plasma cystatin C

Proportional hazards assumption was not violated in any of the models

*DMA* dimethylamine, *ADMA* asymmetric dimethylarginine, *SDMA* symmetric dimethylarginine, *BMI* body mass index, *eGFR* estimated glomerular filtration rate, *HDL* high-density lipoprotein

## Discussion

Protein-arginine dimethylation is a major post-translational modification (PTM) (Scheme 1). Arginine-moieties of numerous proteins in histones and other molecular structures are methylated by PRMT in many tissues and cells, notably in red blood cells (Bollenbach et al. 2020a; b). The physiological importance of arginine-dimethylated proteins is currently of major scientific interest (Greer and Shi 2012; Blanc and Richard 2017; Peng and Wong 2017; Beltran-Alvarez et al. 2011, 2014, 2015; Samuel et al. 2021; Sirover 2021). Besides their protein-related functions, arginine-dimethylated proteins are precursors of MMA, ADMA and SDMA. These methylated arginine metabolites are inhibitors of NOS activity (Tsikas et al. 2000) and possess additional not yet elucidated, presumably NO-independent biological functions (Tsikas 2017; Tsikas et al. 2018; Zewinger et al. 2017).

The enzymes of the Arg/PRMT/DDAH/NO pathways are ubiquitous. The kidney and the liver are mainly responsible for the elimination of ADMA and SDMA (Nijveldt et al. 2002, 2003). SDMA is excreted in the urine virtually without metabolization. ADMA is excreted in the urine in part unchanged and in part (by about 80%) after metabolization to DMA (Achan et al. 2003; Tsikas 2020). High concentrations of circulating ADMA and SDMA are considered as risk factors for cardiovascular disease and renal outcome in chronic kidney disease (Busch et al. 2006). ADMA, DMA and SDMA are sporadically measured in human urine (Tsikas et al. 2007). In contrast to circulating ADMA, low urinary ADMA concentrations were found to be a predictor of mortality risk in patients with coronary artery disease (Wolf et al. 2012), underlying the importance of the kidney and urinary ADMA in clinical settings. Because of the particular importance of the kidney in Arg/PRMT/DDAH/NO pathways, chronic renal disease, end-stage renal disease and kidney transplantation provide valuable opportunities to study the relative contribution of renal Arg/PRMT/DDAH/NO pathways to disease development, progression and outcome in renal transplant recipients (RTR) and kidney donors in humans.

In the context of a previously described study (van den Berg et al. 2012, 2013, 2014), which is part of a larger prospective cohort study of RTR in northern Netherlands, we have measured in plasma and urine samples ADMA, SDMA, homoarginine and guanidinoacetate (Frenay et al. 2015a, b; Kayacelebi et al. 2017; Hanff et al. 2019; Said et al. 2019a, b). Homoarginine and guanidinoacetate are formed from arginine by the catalytic action of arginine:glycine amidinotransferase (AGAT; EC 2.1.4.1) in kidney and liver (Tsikas and Wu 2015). In the present study, we measured the urinary excretion of DMA, the major urinary metabolite of ADMA, in RTR and donors and investigated its association with mortality. In addition, we performed similar analyses for DMA + ADMA, DMA + ADMA + SDMA, and (DMA + ADMA)/SDMA. These composites were found to be useful measures of the whole-body asymmetric, asymmetric and symmetric dimethylation, and of the balance of asymmetric-to-symmetric dimethylation of arginine residues in proteins (Bollenbach et al. 2020a; b). After successful re-evaluation of a previously reported validated GC–MS method for DMA (Tsikas et al. 2007), we applied this method in the current study. The results of the re-validation and the concomitantly processed QC samples underline the high analytical reliability of the GC–MS method for determining urinary DMA concentrations.

Clinical and biochemical parameters in the urine of the donors were measured before and on average after 1.6 months after kidney donation (Table S1). Systolic and diastolic blood pressure decreased significantly by 4% and 3%, respectively. The greatest change was observed in renal function: eGFR decreased almost by 50%. In contrast to ADMA and SDMA (PRMT/DDAH metabolites) and to homoarginine and guanidinoacetate (AGAT pathway) (Frenay et al. 2015a, b; Kayacelebi et al. 2017; Hanff et al. 2019; Said et al. 2019a, b), donation of one kidney by the healthy donors revealed only a small, statistically insignificant decrease in urinary DMA excretion (by − 9%). These observations suggest a rather minor contribution of the kidneys to urinary DMA, but considerable contributions to urinary ADMA and SDMA excretion.

Also in contrast to ADMA, the excretion rate of DMA was associated with all-cause mortality, but not with cardiovascular or non-cardiovascular mortality in the RTR. Regarding mortality, total protein-arginine dimethylation appears to behave reversely compared to the ratio of asymmetric-to-symmetric protein-arginine dimethylation (a/sPADiMeX): a/sPADiMeX values above 8 seem to be associated with increasing all-cause mortality (70% survival after 6-year follow-up in the highest tertile).

PRMT isoforms use *S*-adenosylmethione (SAM) as methyl-group donor. As RTR diets contain methionine (1.88 g/day), the precursor of SAM, it is reasonable to assume that the RTR of our study were not SAM-deficient.

A possible limitation of our study could be the contribution of additional endogenous and exogenous sources to urinary DMA, including DMA-rich food notably fish (reviewed in Tsikas 2020). Both ADMA and SDMA are also metabolized by alanine:glyoxylate aminotransferase 2 (AGXT2) (Jarzebska et al. 2019) and *N*-acetylases (Rodionov et al. 2016), albeit to a three orders of magnitude lower extent compared to DDAH (Martens-Lobenhoffer et al. 2014).

Strengths of the present study are the reliable measurements of protein-arginine dimethylation, the large sample size of this well-defined cohort, the presence of appropriate controls, the long follow-up and the collection of a wide variety of demographical and laboratory parameters allowing adjustment for many potential confounders. Nonetheless, several limitations of this study need to be addressed. In general, statistical significance in observational studies does not confirm biologic significance. It is unknown whether the relations between protein-arginine dimethylation parameters and mortality are causal or associative. In addition, our study population consisted predominantly of Caucasian individuals, which precludes us from extrapolation of our results to populations of other ethnicities. Furthermore, the possibility of residual confounding remains. Lastly, we did not have data on plasma SDMA and plasma DMA, so we were unable to perform analyses on circulating concentrations of SDMA and DMA.

In conclusion, we found that lower DMA excretion rates were associated with higher all-cause mortality, yet not with cardiovascular mortality in RTR. In the healthy donors, kidney donation was associated with considerable decreases in ADMA (by − 39%, *P* < 0.0001) and SDMA (by − 21%, *P* < 0.0001) excretion rates, yet there was a smaller but not significant change in DMA (by − 9%, *P* = 0.226) excretion rate. Our results suggest that protein-arginine dimethylation is altered in RTR compared to healthy kidney donors and that it is pronouncedly shifted from symmetric to asymmetric arginine-dimethylation, with whole-body protein-arginine dimethylation being almost unaffected.

## Supplementary Information

Below is the link to the electronic supplementary material.

Supplementary file1 (DOCX 514 KB)

## Abbreviations

ADMA: Asymmetric dimethylarginine
BMI: Body mass index
BSA: Body surface are
CVD: Cardiovascular disease
CI: Confidence interval
DDAH: Dimethylarginine dimethylaminohydrolase
DMA: Dimethylamine
GC–MS: Gas chromatography–mass spectrometry
HR: Hazard ratio
IQR: Interquartile range
NO: Nitric oxide
NOS: Nitric oxide synthase
PADiMe: Protein-arginine dimethylation
PADiMeX: Protein-arginine dimethylation indices
PRMT: Protein-arginine methyltransferase
PTM: Post-translational modification
RTR: Renal transplant recipients
SDMA: Symmetric dimethylarginine
