# Supplementary material for: Whole-body arginine dimethylation is associated with all-cause mortality in adult renal transplant recipients
Source: Amino Acids. 2021 Mar 2;53(4):541–54. doi: 10.1007/s00726-021-02965-1 (PMC8107162; doi:10.1007/s00726-021-02965-1)
Supplement: Supplementary file 1 — Supplementary file1 (DOCX 514 KB) [file 726_2021_2965_MOESM1_ESM.docx]

**Supplement to:**

**Whole-body arginine dimethylation is associated with all-cause mortality in adult renal transplant recipients**

Adrian Post^1,a,*^, Alexander Bollenbach^2,a^, Stephan J.L. Bakker^1,b^, Dimitrios Tsikas^2,b,*^

^1^ Division of Nephrology, Department of Internal Medicine, University Medical Center Groningen and University of Groningen, Groningen, The Netherlands

^2^ Core Unit Proteomics, Institute of Toxicology, Hannover Medical School, Carl-Neuberg-Str. 1, 30625 Hannover, Germany

Table S1. Anthropometric and clinical characteristics of the healthy donors before and after kidney donation

| **Variables** | | **Pre-donation** | **Post-donation** | **Difference (%)** | ***P*** |
| --- | --- | --- | --- | --- | --- |
| **Demographics** | |  |  |  |  |
|  | Age (years) | 52.1 ± 9.9 | 52.7 ± 10.0 | 1.21 ± 0.80 | <0.001 |
|  | Male [*n* (%)] | 59 (45.4) | n/a | n/a | n/a |
| **Time after donation (months)** | | n/a | 1.64 [1.61–1.87] | n/a | n/a |
| **Body proportions** | |  |  |  |  |
|  | Weight (kg) | 79.8 ± 12.4 | 79.2 ± 13.0 | -0.92 ± 3.90 | 0.01 |
|  | BMI (kg/m^2^) | 26.1 ± 3.4 | 25.9 ± 3.3 | -0.51 ± 5.04 | 0.14 |
|  | BSA (m^2^) | 1.95 ± 0.19 | 1.94 ± 0.20 | -0.40 ± 1.81 | 0.02 |
| **Cardiovascular parameters** | |  |  |  |  |
|  | SBP (mmHg) | 128 ± 14 | 122 ± 12 | -4.0 ± 8.2 | <0.001 |
|  | DBP (mmHg) | 78 ± 8 | 75 ± 9 | -3.1 ± 10.8 | <0.001 |
| **Blood parameters** | |  |  |  |  |
|  | HbA_1c_ (%) | 5.5 ± 0.3 | 5.5 ± 1.0 | 0.3 ± 5.3 | 0.82 |
| **Urinary excretion** | |  |  |  |  |
|  | Urea (mmol/24 h) | 417 ± 126 | 394 ± 113 | 0.8 ± 39.1 | 0.06 |
|  | Creatinine (mmol/24 h) | 13.5 ± 4.4 | 12.8 ± 4.1 | -1.1 ± 27.9 | 0.07 |
| **Renal function** | |  |  |  |  |
|  | eGFR (mL/min/1.73 m^2^) | 96.5 ± 13.9 | 49.5 ± 11.1 | -48.5 ± 8.8 | <0.001 |

Abbreviations: n/a, not applicable

Table S2. Correlation coefficients (*r*) and *P*-values from Spearman correlation analyses between the excretion rates (µmol/24 h) or molar ratio values in the urine of the 76 healthy kidney donors pre-donation (pre) and post-donation (post)

| Measure | DMA | | ADMA | | SDMA | | DMA+ADMA | | DMA+ADMA+SDMA | |
| --- | --- | --- | --- | --- | --- | --- | --- | --- | --- | --- |
| Kidney donation | pre | post | pre | post | pre | post | pre | post | pre | post |
| ADMA | *r*=0.584 *p*<0.0001 | *r*=0.507  *p*<0.0001 |  |  |  |  |  |  |  |  |
| SDMA | *r*=0.622  *p*<0.0001 | *r*=0.775  *p*<0.0001 | *r*=0.774 *p*<0.0001 | *r*=0.550 *p*<0.0001 |  |  |  |  |  |  |
| DMA+ADMA | *r*=0.692  *p*<0.0001 | *r*=0.998  *p*<0.0001 | *r*=0.581 *p*<0.0001 | *r*=0.554 *p*<0.0001 | *r*=0.478 *p*<0.0001 | *r*=0.777 *p*<0.0001 |  |  |  |  |
| DMA+ADMA+SDMA | *r*=0.980 *p*<0.0001 | *r*=0.995  *p*<0.0001 | *r*=0.681 p<0.0001 | *r*=0.548 *p*<0.0001 | *r*=0.744 *p*<0.0001 | *r*=0.817 *p*<0.0001 | *r*=0.703 *p*<0.0001 | *r*=0.996 *p*<0.0001 |  |  |
| (DMA+ADMA)/SDMA | *r*=-0.140 *p*=0.23 | *r*=0.133  *p*=0.33 | *r*=-0.369 *p*=0.001 | *r*=0.037 *p*=0.75 | *r*=-0.701 *p*<0.0001 | *r*=-0.445 *p*<0.0001 | *r*=0.214 *p*=0.06 | *r*=0.119 *p*=0.31 | *r*=-0.264 *p*=0.02 | *r*=0.060 *p*=0.61 |

Table S3. Correlation coefficients (*r*) and *P*-values from Spearman correlation analysis between the excretion rates (µmol/24 h) or molar ratio values in the urine of the 691 renal transplant recipients

| Measure | DMA | ADMA | SDMA | DMA+ADMA | DMA+ADMA+SDMA |
| --- | --- | --- | --- | --- | --- |
|  | Spearman correlation coefficient | | | | |
| ADMA | *r*=0.399 |  |  |  |  |
| SDMA | *r*=0.558 | *r*=0.573 |  |  |  |
| DMA+ADMA | *r*=0.994 | *r*=0.484 | *r*=0.590 |  |  |
| DMA+ADMA+SDMA | *r*=0.984 | *r*=0.512 | *r*=0.664 | *r*=0.993 |  |
| (DMA+ADMA)/SDMA | *r*=0.247 | *r*=-0.236 | *r*=-0.595 | *r*=0.119 | *r*=0.119 (*p*=0.0018) ^a^ |

^a^ *The p values of all other correlations were p<0.0001*


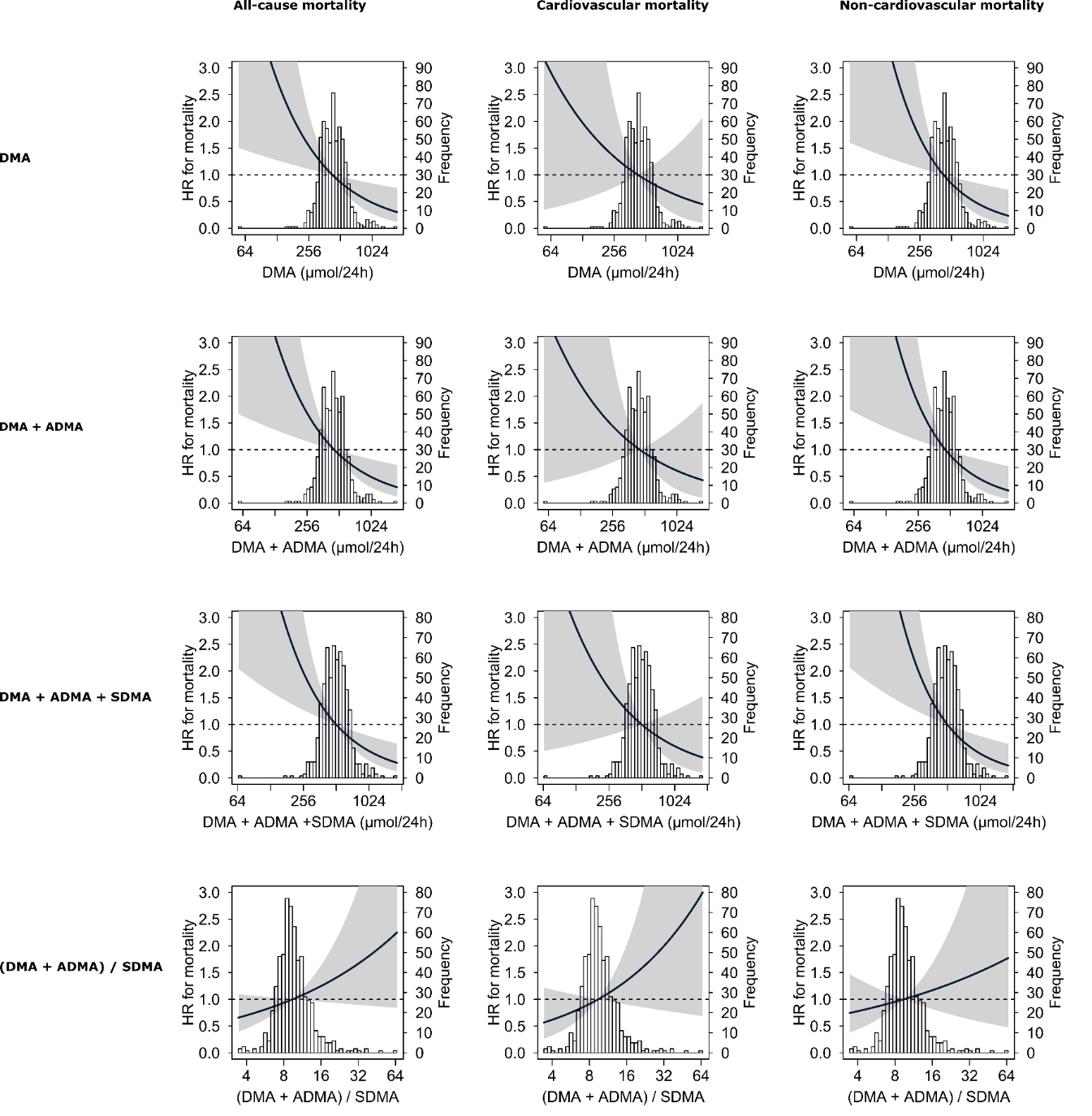


Figure S1. Hazard ratio (HR) curves for urinary protein-arginine dimethylation parameters and mortality outcomes.


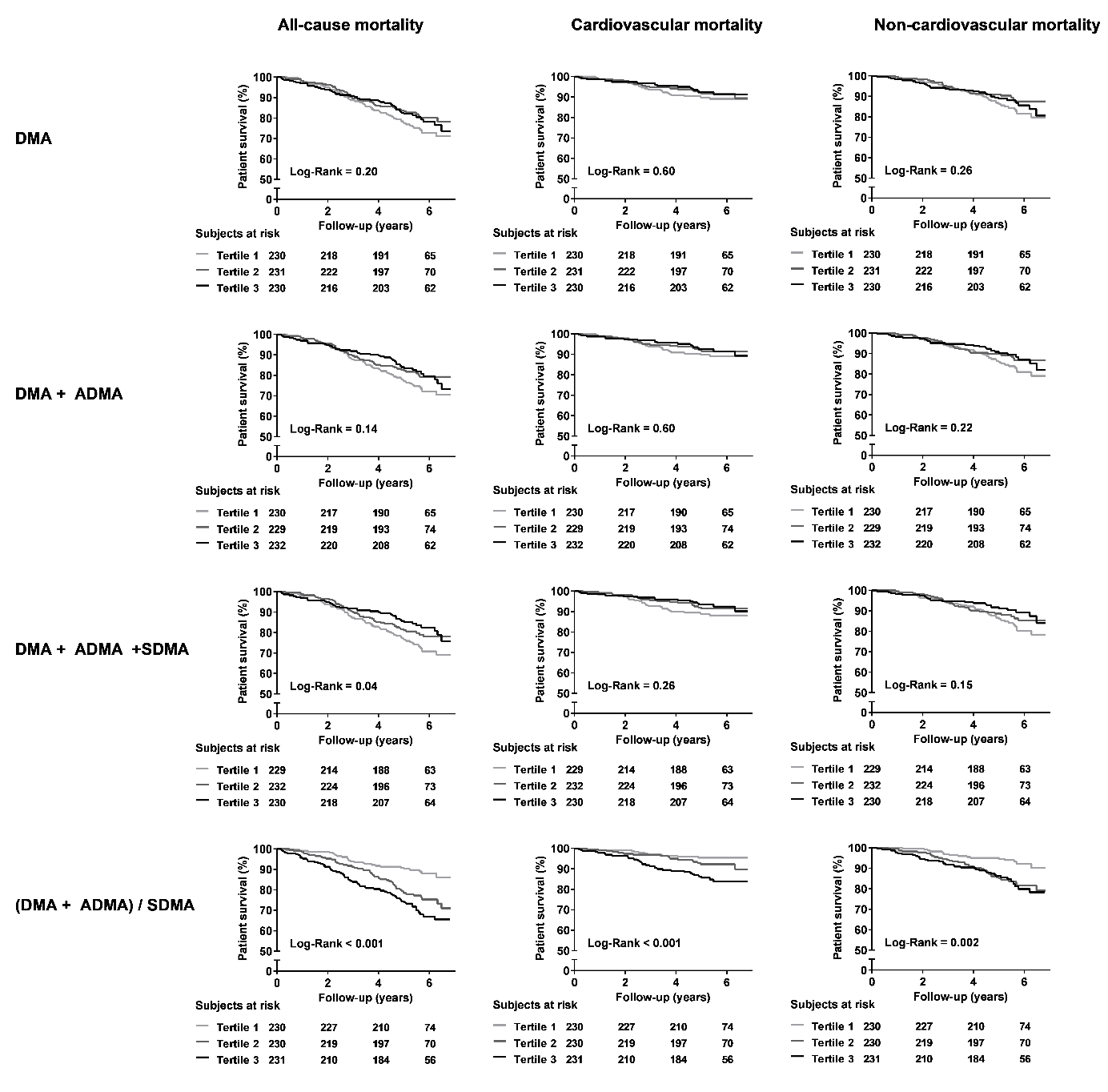


Figure S2. Kaplan-Meier curves for urinary protein-arginine dimethylation parameters and mortality outcomes.

| Table S4. Associations of plasma ADMA with urinary excretion of ADMA, DMA and DMA + ADMA + SDMA | | | | |
| --- | --- | --- | --- | --- |
| **Model** | **AMDA excretion** | | **DMA excretion** | |
|  | **Std. β** | **P-value** | **Std. β** | **P-value** |
| Model 1 | -0.07 | 0.06 | 0.02 | 0.64 |
| Model 2 | -0.09 | 0.02 | -0.02 | 0.72 |
| Model 3 | -0.09 | 0.03 | -0.01 | 0.92 |
| Model 4 | 0.36 | <0.001 | -0.01 | 0.75 |
| Plasma SDMA is the dependent variable in each of the analyses. Model 1: Crude. Model 2: Adjusted for age and sex. Model 3: As model 2, additionally adjusted for BMI. Model 4: As model 3, additionally adjusted for estimated glomerular filtration rate. | | | | |
